# Conserved regulation of autophagosome-lysosome fusion through YKT6 phosphorylation

**DOI:** 10.1080/27694127.2023.2210946

**Published:** 2023-05-10

**Authors:** Pablo Sánchez-Martín, Claudine Kraft

**Affiliations:** a Institute of Biochemistry and Molecular Biology, ZBMZ, Faculty of Medicine, University of Freiburg, Freiburg, Germany; b CIBSS - Centre for Integrative Biological Signalling Studies, University of Freiburg, Freiburg, Germany

**Keywords:** ULK1, autophagosome, autophagy, SNARE, YKT6

## Abstract

YKT6 is a SNARE (Soluble N-ethylmaleimide-Sensitive Fusion Protein Attachment Protein Receptor) protein governing membrane fusion events of several cellular organelles. In autophagy, YKT6 is involved in early phagophore formation as well as directly in the fusion process between autophagosomes and the lytic compartment. Recently we showed in yeast, mammalian cells, and nematodes that the function of YKT6 in autophagy can be regulated by phosphorylation. Atg1/ULK1 (Unc-51-like kinase 1)-dependent phosphorylation of YKT6 results in autophagy defects during both early (autophagosome formation) and late (autophagosome-lysosome fusion) steps, ultimately resulting in decreased survival of mammalian cells due to defective stress-induced autophagy. These findings show that not only the function but also the regulation of YKT6 is conserved across species.

## Abbreviations

SNARE, Soluble N-ethylmaleimide-Sensitive Fusion Protein Attachment Protein Receptor; ULK1, Unc-51-like kinase 1; STX17, Syntaxin17; SNAP29, Synaptosome Associated Protein 29; VAMP8, Vesicle Associated Membrane Protein 8; STX7, Syntaxin7; LAP, LC3-associated phagocytosis.

In eukaryotic organisms that contain membrane-delimited organelles, a machinery enabling the transport across and fusion of membranes is required. This allows for instance the secretion of extracellular vesicles, intracellular trafficking, and the delivery of cargo to lytic compartments during autophagy. Among the proteins involved in membrane fusion, SNARE (Soluble N-ethylmaleimide-Sensitive Fusion Protein Attachment Protein Receptor) proteins play a crucial role and are the minimum requirement to enable this event to occur. In brief, a tight bundle of four alpha-helices is formed by four SNARE modules that reside on the surfaces of the opposing membranes. The energy released during the formation of the bundle drives the mixing of the adjacent membranes and their fusion. In order to understand the fusion process of autophagosomes with lytic compartments, it thus has been crucial to identify the SNARE proteins that are involved, to which membrane they localize, and how their function is regulated.

**Figure 1.**
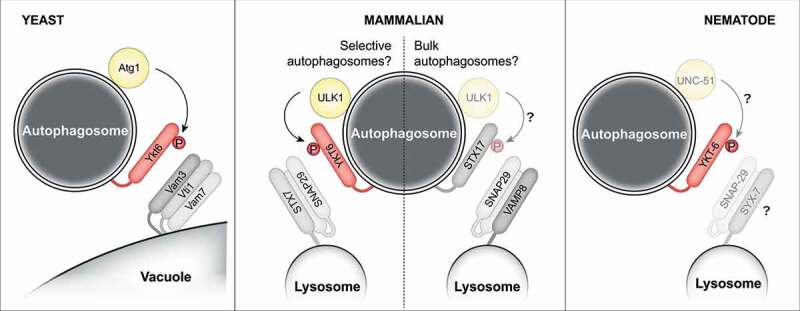
Model depicting the potentially conserved regulation of autophagosome-lysosome fusion from yeast to mammals. See text for details.

In recent years, the main SNARE complexes involved in the fusion of autophagosomes with late endosomes/lysosomes in mammals have been identified: STX17 (Syntaxin17)-SNAP29 (Synaptosome Associated Protein 29)-VAMP8 (Vesicle Associated Membrane Protein 8) and YKT6-SNAP29-STX7 (Syntaxin7) (Figure 1). Both complexes have a similar membrane distribution, with YKT6 or STX17 located on the surface of the autophagosome, while SNAP29 is soluble in the cytosol, and VAMP8 and STX7 sitting on the lysosome surface. Although mammals possess two SNARE complexes that can drive autophagosome-lysosome fusion, a single complex is sufficient for this fusion step in budding yeast, which only possesses the Ykt6-containing SNARE set. However, the mammalian two complexes are not completely redundant, as the loss of only SXT17 or YKT6 drastically impairs autophagy. One possibility is that each group of SNAREs acts on different types of autophagosomes (bulk or selective) or different types of selective autophagy. This would require a fine regulation of STX17 and YKT6, for their targeting to specific autophagosomal membranes as well as their local function. It should be noted that YKT6 in *Drosophila* might operate in a different manner than in yeast, mammals, and *Caenorhabditis elegans*, as it has been proposed to play a regulatory role on the STX17 module rather than being part of a parallel-acting SNARE complex.

Previously, we and others found that yeast Ykt6 function is regulated by Atg1-dependent phosphorylation, preventing the interaction with other SNARE proteins and thus autophagosome-vacuole fusion until the autophagosome is completed. This phospho-regulation likely takes place on membrane-bound Ykt6 at the surface of the phagophore and does not affect its membrane recruitment. Thus, we asked if the function of YKT6 in fusion is regulated similarly to mammals. The interrelationships between the two mammalian SNARE complexes are still not fully understood, and the STX17-containing complex has been found to be stimulated by ULK1 (Unc-51-like kinase 1), the mammalian ortholog of Atg1, a notion that is in contrast to the inhibitory function of Atg1 on yeast Ykt6. Moreover, phospho-regulation of mammalian YKT6 has previously been reported, but the analyzed phosphorylation sites predominantly affected the recruitment of YKT6 to membranes rather than its local function, which could also result in indirect effects on the autophagy flux. When analyzing the regulation of mammalian YKT6, we found that phosphorylation on threonine 156 results in defects in autophagosome formation and a block of autophagosome-lysosome fusion. Although the mechanism causing autophagosome formation defects remains unknown, the fusion defects are caused by a defective interaction between YKT6 and SNAP29 [1]. Similar to the regulation of yeast Ykt6, the phosphorylation takes place in the SNARE domain, and this site is a target of ULK1, at least *in vitro*. This conserved mechanism is different from the previously described phosphorylation events for mammalian YKT6, as it does not affect the membrane localization of YKT6. Rather, YKT6 phosphorylation by ULK1 regulates its fusogenic activity after YKT6 has been recruited to the autophagosomal membrane. Such regulation allows the local control of YKT6 function only on autophagosomes, without affecting its function in other cellular processes.

These findings furthermore point out that early regulators of autophagy, such as Atg1/ULK1, can also play a role in the late steps of the process. However, such additional, later roles are often overseen as they are masked during analyses, due to the early defect the mutation or depletion of such early regulators cause. Therefore, *in vitro* reconstitution of late steps of such processes is of enormous help to uncover the role of early regulators also during the late stages of a specific step of autophagy. For both yeast and mammalian cells we have reconstituted autophagosome-vacuole/lysosome fusion *in vitro*, which allowed the precise dissection of YKT6 function and its phospho-regulation during this specific step.

To further study the conserved regulation of YKT6 across species, we analyzed the role of YKT-6 in *C. elegans*, which had scarcely been studied. Depletion of YKT-6 resulted in a defect in autophagosome formation also in worms. For *C. elegans*, the development of an *in vitro* system to study autophagosome-lysosome fusion is very difficult, if not impossible. However, worms utilize LC3-associated phagocytosis (LAP) for the removal of apoptotic cells in the germline. During LAP, phagosome formation happens independently of the autophagy machinery but their fusion with lysosomes requires many of the proteins that are also known to be involved in autophagosome-lysosome fusion. Therefore, phagosomes likely utilize the same fusion machinery as autophagosomes. Studying the fusion of phagosomes with lysosomes in worms thus allows the separate analysis of late steps of autophagy *in vivo* and the specific analysis of the regulation of the phagosome-lysosome fusion machinery. This system allowed us to uncover that a phospho-mimetic mutant of YKT-6 in worms showed a similar defect in this fusion process as observed in yeast and mammals, suggesting that the function and regulation of YKT-6 are also conserved in *C. elegans*. It remains to be further tested if UNC-51, the Atg1/ULK1 orthologous kinase, is also responsible for this phosphorylation in worms, how and when the phosphorylation takes place and which lysosomal SNARE proteins interact with YKT-6 during autophagosome-lysosome fusion. STX7 and SNAP29, which are the mammalian partners of YKT6 during autophagosome-lysosome fusion, have also orthologs in *C.elegans*. The second mammalian set of SNAREs mediating this fusion process in mammals, STX17, SNAP29, and VAMP8, are also conserved in worms and can, at least *in vitro*, form a SNARE complex. This might suggest that worms employ also two sets of SNARE complexes for autophagosome-lysosome fusion.

Another observation was that loss of YKT6 function resulted in impaired mitophagy, the selective removal of damaged mitochondria by autophagy. Loss of STX17, however, does not affect mitophagy. Bulk and selective autophagosomes can differ in size, with bulk autophagosomes being mostly relatively large and selective autophagosomes being often smaller, depending on the cargo. YKT6 and STX17 utilize distinct mechanisms for their attachment to the autophagosomal membrane. YKT6 membrane binding is regulated through lipid modifications, which could allow preferential binding to membranes with high curvature, whereas STX17 has a transmembrane domain that might insert easier in larger membranes with low curvature. This would be in line with the idea that the YKT6-containing set of SNAREs preferentially acts on selective autophagosomes, while STX17 acts on bulk autophagosomes.

One last point of interest is if the two complementary but not redundant sets of SNARE complexes are regulated in a similar manner. For instance, it has been shown that ULK1 interacts with STX17, enhancing autophagosome-lysosome fusion, which is in contrast with its inhibitory role on YKT6. If this increase in fusion turns out to be a consequence of direct phosphorylation of STX17 by ULK1, it could indicate the existence of a regulatory switch by which ULK1 phosphorylation of the autophagosomal SNAREs can induce fusion when acting on STX17, but inhibit fusion when acting on YKT6. Such a reciprocal regulation might happen on different membrane structures decorated with different SNARE proteins and might thereby be able to discriminate between selective and bulk autophagosomes. As both SNARE complexes have been also found to partially colocalize on the same autophagosomal structures, ULK1 could allow only the activity of one but not the other SNARE complex. If such regulation on STX17 takes place and what the role of a reciprocal ULK1-dependent regulation could be, remains to be addressed.

Further work is required to elucidate the precise regulation of autophagosome-lysosome fusion in bulk and selective autophagy pathways, and its conservation among organisms.
